# The role of molecular diagnostics in pleural empyema: insights from a 4-year analysis of microbial trends and antimicrobial use

**DOI:** 10.1099/jmm.0.002098

**Published:** 2025-11-28

**Authors:** Amy Carroll, Emma McGuire, Pietro Coen, Paul Grant, Shara Palanivel

**Affiliations:** 1 Department of Clinical Microbiology, University College London Hospitals NHS Foundation Trust, London, UK; 2 Infection Prevention & Control Office, University College London Hospitals NHS Foundation Trust, London, UK; 3 Department of Molecular Pathology, Health Services Laboratories, London, UK

**Keywords:** antibiotics, antimicrobial stewardship, empyema, molecular diagnostics, pleural infection, thoracic surgery

## Abstract

**Introduction.** Pleural empyema is common but microbiological diagnosis is challenging, often leading to prolonged empiric broad-spectrum antimicrobial use.

**Hypothesis.** 16S rRNA PCR on operative pleural tissue will increase diagnostic yield and improve antibiotic stewardship in patients with surgically managed pleural empyema.

**Aim.** The objective was to characterize the clinical and microbiological characteristics of surgically managed pleural empyema, assess the diagnostic yield of 16S rRNA PCR and determine its impact on antimicrobial use.

**Methodology.** This retrospective study identified patients with pleural empyema at a tertiary thoracic referral unit in North London, UK, between March 2019 and March 2023. Demographic, clinical and microbiological characteristics were collected.

**Results.** One hundred eighty-three patients underwent surgery for pleural empyema at University College Hospital at Westmoreland Street over a 4-year period. Most empyemas were community-acquired, and the main surgical modality was minimally invasive video-assisted thoracoscopic surgery (151/183, 82.5%). Thirty-day all-cause post-operative mortality was 0.6%. Microbial aetiology was ascertained in 47%, with *Streptococcus anginosus* group, *Streptococcus pneumoniae* and anaerobes most frequently identified. Operative pleural tissue 16S rRNA PCR had a high diagnostic yield compared to conventional culture alone (68.2% vs. 26.4%). Post-operatively, antibiotics were prescribed for a median of 14 days (interquartile range 10–18), the majority broad spectrum (169/181, 93.4%). 16S PCR impacted antimicrobial prescribing in one-tenth of cases when used (4/32, 12.5%); however, turnaround times limited its impact.

**Conclusion.** Molecular diagnostics improve bacterial detection and antibiotic stewardship in pleural empyema. Streamlining molecular diagnostics pathways or developing pleural-specific multiplex PCR panels could further enhance management.

## Introduction

Pleural empyema incidence is rising in the UK and continues to be associated with high morbidity and mortality [1,2]. Empyema develops following translocation of bacteria into the pleural space, which can occur through local suppurative processes, haematogenous seeding from distal sites of infection or after thoracic interventions or chest wall trauma [1,3]. The mainstay of management is with medical interventions, including antibiotics, closed chest drainage and intrapleural treatments. For patients with poor response to medical management, surgery is indicated to achieve source control. This involves clearing the pleural cavity through debridement and promoting lung re-expansion [3,4].

Most pleural infections are polymicrobial, usually involving anaerobes as co-pathogens, except for empyema caused by *Streptococcus pneumoniae,* which is typically monomicrobial [5,6]. Accurate microbiological diagnosis is challenging because pleural space cultures have low yield (30–40% positivity), due to rapid sterilization by antibiotics and low culture sensitivity for anaerobes [1,4]. This diagnostic uncertainty often leads to antibiotic overuse. Molecular diagnostics, such as 16S rRNA PCR, could allow earlier optimization of antibiotic therapy. This study aimed to characterize the clinical and microbiological characteristics of surgically managed pleural empyema over a 4-year period (March 2019–March 2023). Secondary objectives included assessing the diagnostic yield of 16S rRNA PCR in culture-negative operative pleural samples, evaluating local prescribing practices, and determining the impact of 16S rRNA PCR results on antibiotic choice and duration.

## Methods

We conducted a retrospective study of all adult patients diagnosed with pleural empyema at University College Hospital at Westmoreland Street between March 2019 and March 2023. University College Hospital at Westmoreland Street is a tertiary referral centre for thoracic surgery serving North London, Hertfordshire, Bedfordshire and Essex as part of University College Hospitals NHS Trust. Data were retrieved from the Trust Electronic Health Record System and Lab Information Management System. This service evaluation project was approved by the local Microbiology Department lead and was exempt from ethical approval as all data were anonymized and collected retrospectively.

Clinical and treatment data were collected, including empyema aetiology, surgical procedures and complications, antibiotic usage and duration and 30-day all-cause post-operative mortality. Empyema cases were classified as hospital-acquired if onset was ≥2 days after hospital admission, within 4 weeks of prior hospitalization, or as a complication of an invasive thoracic procedure. Community-acquired empyema was defined as empyema due to an infection syndrome present in the days to weeks prior to hospital admission. Antibiotic type was defined as the predominant antibiotic prescribed (>50% of total treatment time, including post-discharge). Antibiotics were categorized into narrow- and broad-spectrum groups (see Appendix S1). A significant change in antimicrobial therapy was defined as any adjustment in spectrum, a switch to target-specific pathogens or a change in treatment duration driven solely by 16S rRNA PCR results. Microbiological data were collected for operative samples and any available pre-operative diagnostic samples (including tests prior to transfer).

All microbiological testing was performed at the Health Services Laboratory using standardized protocols based on the UK Standards for Microbiological Investigations. Bacterial identification was performed primarily with matrix-assisted laser desorption/ionization time of flight. Antimicrobial susceptibility testing followed European Committee on Antimicrobial Susceptibility Testing (EUCAST) guidelines. Culture-negative pleural tissue specimens underwent 16S rRNA PCR at the discretion of the treating clinician or infection specialist in cases of suspected pleural infection. The sample was extracted using the EZ1® DSP Virus Kit on a QIAGEN EZ1 Advanced XL (Cat no. 62724; QIAGEN, Hilden, Germany). Tissue samples had an additional protease lysis step prior to extraction. 16S rRNA PCR was performed using the Taq PCR Master Mix Kit (Cat no. 201443; QIAGEN, Hilden, Germany) according to the manufacturer’s instructions and the primers published by Harris *et al*. [7]. PCR amplicons underwent cycle sequencing using the BigDye Termination v3.1 cycle sequencing kit (Cat no. 4337455; Applied Biosystems, Foster City, CA, USA) according to the manufacturer’s instructions. Forward and reverse FASTA sequences were aligned using SeqScape software (Applied Biosystems, Foster City, CA, USA) before being searched against SepsiTest™-blast or National Center for Biotechnology Information (NCBI) blastn for identification and comparison. A query coverage ≥95% and sequence identity ≥99% were considered acceptable to identify the bacterium to the species level. However, where the percentage of identical base pairs was <95% or if the value was ≥95%, but multiple species were identified within a genus, then identification was reported to the genus level only. Sequencing was considered a failure when small DNA quantities resulted in low-quality sequencing data that did not meet the criteria for identifying bacteria to the genus and/or species level. When this occurred, the PCR and sequencing were repeated but were reported as a failure if there was no improvement in the sequencing data quality. Microbiology results were deemed to be clinically significant based on infection team documentation or retrospective review by study authors.

Data were analysed using STATA [14.2] (StataCorp, College Station, Texas). The Kruskal–Wallis test was used to assess associations between continuous and discrete variables. Bivariate comparisons were performed using chi-squared or Fisher’s exact tests, as appropriate.

## Results

### Baseline characteristics

Over the 4-year study period, 183 patients underwent surgical management of pleural empyema. The cohort was predominantly male (78.1%), with a median age of 60 years (Interquartile range (IQR) 49–72) (Table 1). In most cases, empyema was right-sided (105/183; 57.4%), secondary to pneumonia (138/183, 75.4%) and community-acquired (151/183, 82.5%). Among the 32 hospital-acquired cases (17.5%), most developed following hospital-acquired pneumonia (15/32, 46.9%) or invasive thoracic interventions (12/32, 37.5%). A large proportion of patients were transferred from other hospitals (158/183, 86.3%), and almost all (181/183, 98.9%) received antibiotic therapy prior to surgery.

**Table 1.** Demographic and clinical characteristics for surgically managed pleural empyema cases at University College Hospital at Westmoreland Street, March 2019–March 2023 (*n*=183)

Characteristic	
Demographics	
Male, *n* (%)	143 (78.1)
Age, median (IQR)	60 (49–72)
Clinical characteristics, *n* (%)	
Right-sided empyema	105 (57.4)
Community-acquired	151 (82.5)
Hospital-acquired	32 (17.5)
Empyema aetiology, *n* (%)	
Community-acquired	
Para-pneumonic	138 (75.4)
Post-traumatic	5 (2.7)
Deep neck space or odontogenic infection	2 (1.1)
Aspiration	4 (2.2)
No clear cause	2 (1.1)
Hospital-acquired	
Para-pneumonic	15 (8.2)
Post-thoracic intervention	12 (6.6)
Deep neck space or odontogenic infection	2 (1.1)
Hydropneumothorax	2 (1.1)
Post-traumatic	1 (0.5)
Referral mechanism, *n* (%)	
Inpatient transfer from a different hospital	158 (86.3)
Inpatient at the same hospital	15 (8.2)
Elective admission from the community	10 (5.5)

### Clinical outcomes

Clinical outcomes are summarized in Table 2. Mortality was low, with only one death (0.6%) within 30 days of surgery. The patient was an elderly individual with community-acquired empyema caused by *Streptococcus anginosus* group infection who died after being transferred back to the referring hospital. The cause of death was not clear from available records.

**Table 2.** Microbiological diagnosis, antibiotic usage and clinical outcomes for cases of surgically managed pleural empyema (*n*=183)

Characteristic	
Microbiological diagnosis, *n/N* (%)	
Total with microbiological diagnosis	86/183 (47.0)
Total with positive pre-operative sample*	42/183 (23.0)
Total with positive operative pleural tissue†	63/182 (34.6)
Total culture positive/tested	48/182 (26.4)
Total 16S PCR positive‡/tested	21/32 (65.6)
16S PCR positive‡/tested culture negative samples	15/22 (68.2)
Antibiotic usage	
Antibiotics prior to surgery, *n/N* (%)	181/183 (98.9)
Antibiotics after surgery, *n/N* (%)	181/183 (98.9)
Broad spectrum, *n/N* (%)	169/181 (93.4)
Narrow spectrum, *n/N* (%)	12/181 (6.6)
Post-operative duration of antibiotics, median (IQR)	14 (10–18)
16S PCR result impacted prescribing, *n/N* (%)	4/32 (12.5)
Clinical outcomes	
30-day all-cause mortality, *n/N* (%)	1/183 (0.06)
Minimally invasive surgical approach by VATS, *n/N* (%)	151/183 (82.5)
LOS§: total, median (IQR)	17 (13–24)
LOS: operation to discharge or transfer¶, median (IQR)	5 (4–9)

*Diagnostic pre-operative sample types include: pleural fluid (*n*=25), blood culture (*n*=11), urinary pneumococcal antigen (*n*=4) and sputum culture (*n*=2).

†Pleural tissue sample not received in laboratory for one patient.

‡Positive samples detecting relevant organism including one case with positive species-specific PCR, not including samples positive by 16S PCR but unable to sequence (*n*=5).

§Including time at referring hospital.

¶Transfer back to referring hospital (*n*=11).

LOS, length of stay; VATS, video-assisted thoracoscopic surgery.

Most empyema cases were managed by video-assisted thoracoscopic surgery (VATS) drainage and debridement, with or without decortication, depending on operative findings (151/183, 82.5%). A minority underwent an open surgical approach (32/183, 17.5%), evenly split between planned procedures (15/32) and conversions from VATS (17/32). Repeat surgical intervention was required in 13 patients (7.1%). The median length of hospital stay was 17 days (IQR 13–24), with a median postoperative stay of 5 days (IQR 4–9). Hospital-acquired empyema was associated with significantly longer postoperative stays compared to community-acquired cases (median 8 vs. 5 days, *P*=0.01), as was having a microbiological diagnosis compared to no diagnosis (median 7 vs. 5 days, *P*<0.01). Open surgery was not associated with prolonged postoperative stays compared to VATS (median 6.5 vs. 5 days, *P*=0.27).

### Microbiological outcomes

A microbiological diagnosis was made in 47% of cases (86/183), combining preoperative and operative microbiological samples (Table 2). Operative pleural tissue culture had a diagnostic yield of 25.8% (47/182). Broad-range 16S rRNA PCR was performed in 17.5% of cases (32/182), with a diagnostic yield of 65.6% (21/32). Among culture-negative pleural tissue samples, 16S PCR provided a diagnostic result in 68.2%(15/22) of cases (*S. pneumoniae n*=7, *S. anginosus* group *n*=5, *Streptococcus pyogenes n*=1, anaerobes *n*=2) (Table 3). PCR usage was higher in the latter 2 years of the study period. Species-specific PCR was used in one case, where 16S PCR was positive but unable to sequence and subsequent species-specific PCR was positive for *S. pneumoniae*.

**Table 3.** Microbiological characteristics of surgically managed pleural empyema cases (*n*=183)

Bacteria	Pre-operative sample (*n*=183)	Pleural tissue culture (*n*=182)*	Pleural tissue 16S PCR (*n*=32)	16S PCR positive, culture negative (*n*=15)	Total (*n*=183)
No organism identified	–	–	–	–	97 (53.0)
Diagnostic results	42	47	21	15	86 (47.0)
*Staphylococcus aureus*	7	8	–	–	11 (6.0)
*S. anginosus* group	7	14	7	5	23 (12.6)
*S. pneumoniae*	8	2	8	7	15 (8.2)
Other streptococci†	5	4	1	1	9 (4.9)
*Enterococcus* spp.	–	1	–	–	1 (0.5)
*Haemophilus influenzae*	2	–	–	–	2 (1.1)
*Enterobacterales*	3	1	1	–	4 (2.2)
*Pseudomonas aeruginosa*	1	1	–	–	1 (0.5)
Mixed culture/bacterial target‡, §	4	9	1	–	9 (4.9)
Anaerobes§	5	7	3	2	11 (6.0)

NB: Positive results in multiple columns for some patients (e.g. positive pre-operative samples and positive pleural tissue culture/16S PCR) so row sum does not equal the row total.

*Pleural tissue sample not received in laboratory for one patient.

†Other streptococci consist of four *S. pyogenes*, four *Streptococcus mitis* group, two *Streptococcus dysgalactiae* and one *Streptococcus salivarius*.

‡Mixed bacterial 16S PCR was defined as any report with mixed sequence results or when more than one bacterial target detected. Mixed bacterial culture was defined as any culture report where >1 species isolated was deemed to be significant.

§Anaerobes identified as part of mixed or monomicrobial bacterial culture/16S PCR consist of six *Schaalia* species (five *Schaalia meyeri* and one *Schaalia odontolytica*), two *Actinomyces* species (one *Actinomyces oris* and one with no subspecies reported), three *Fusobacterium* species, two *Eikenella corrodens*, one *Aggregatibacter aphrophilus*, one *Alloscardovia omnicolens*, one *Lactobacillus gasseri*, one *Prevotella* species, one *Parvimonas micra*, one *Mycoplasma faucium* and one unspeciated anaerobe.

The most frequently identified pathogens overall were streptococci (47/183, 25.7%), particularly the *S. anginosus* group (*n*=23 monomicrobial infections, *n*=4 polymicrobial with anaerobes) and *S. pneumoniae* (*n*=15, all monomicrobial) (Table 3). Anaerobes were also frequently identified (*n*=11 monomicrobial, *n*=6 polymicrobial). *Staphylococcus aureus* isolates (*n*=11, 6.0%) were all methicillin-sensitive. Hospital-acquired empyema was more frequently associated with *S. aureus* (15.6% vs. 4.0%, *P*=0.02) and polymicrobial infections (15.6% vs. 2.6%, *P*=0.05), while *S. pneumoniae* was strongly associated with community-acquired empyema (9.9% vs. 0%, *P*=0.04).

### Antibiotic usage

Pre-operative and post-operative antibiotics were administered in almost all cases (181/183, 98.9%). Post-operatively, broad-spectrum antibiotics were frequently used (169/181, 93.4%), mainly co-amoxiclav (113/181, 62.4%). Narrow-spectrum agents were used in only 12 cases with confirmed monomicrobial diagnoses. Postoperative antibiotic duration was unavailable in 11 cases due to patient transfers. The median postoperative antibiotic course was 14 days (IQR 10–18). Antibiotics were stopped immediately after surgery in two cases due to clinical stability and prolonged pre-operative courses. A positive microbiological diagnosis was associated with longer postoperative antibiotic duration (median 16 vs. 11 days, *P*<0.01).

16S rRNA PCR results influenced antimicrobial prescribing in 12.5% of cases (4/32), mainly narrowing of the antibiotic spectrum. However, only one-third of positive PCR results (7/21, 33.3%) were available before hospital discharge. The median time from surgery to positive 16S PCR result was 10 days (*n*=21, IQR 8–13). Negative 16S PCR results had no observed impact on antibiotic prescribing practices.

## Discussion

This single-centre retrospective cohort study of surgically managed empyema cases demonstrates the high diagnostic yield of 16S PCR on operative pleural tissue samples, helping differentiate between infection due to *S. pneumoniae* (monomicrobial) and *S. anginosus* group and anaerobes (often polymicrobial). We observed some positive antimicrobial stewardship impacts (12.5% narrowed spectrum among cases where PCR was used); however, there is scope for improvement through streamlining molecular diagnostics pathways or developing rapid syndromic PCR panels.

Our findings align with previous studies regarding demographics, clinical characteristics and microbial aetiology [1,2, 8, 9]. Our cohort predominantly underwent minimally invasive thoracic surgery, with 82.5% managed by VATS. Clinical outcomes were excellent with a low 30-day mortality of 0.6%. Pathogens were identified in 47% overall, with *S. anginosus* group and anaerobes most frequently identified, consistent with previous studies and reinforcing the important role oral pathobionts play in pleural infection [1,5, 6, 8]. Community-onset infections due to pathogens from the human oral microbiota, referred to as oral-type pleural infections, are thought to occur due to haematogenous seeding from the oral cavity [5]. In our study, community-acquired empyema accounted for the majority (82.5%) of cases, and this was strongly associated with identification of *S. pneumoniae*. In contrast, hospital-acquired empyema was associated with *S. aureus* and polymicrobial infection, consistent with the literature [1,6, 8].

Compared to a prior study at our centre conducted from 1999 to 2010, we observed an increased proportion of streptococcal and anaerobic pleural infections, likely due to advancements in molecular diagnostics and evolving trends in pneumococcal infection [1,9,11]. Notably, the proportion of empyema due to *S. pneumoniae* in our cohort was relatively stable (between 2.4 and 7.1%) during the first 3 years of the study period, with a marked increase in 2022–2023 – 14.3% after the Severe acute respiratory syndrome coronavirus 2 (SARS-CoV-2) Coronavirus disease 2019 (COVID-19) pandemic. In the UK, there was an increase in the incidence of adult pneumococcal pneumonia between 2013 and 2018, coinciding with an increase in the incidence of invasive pneumococcal disease (IPD) in older adults over a similar period [10,11]. Studies attributed this change to the persistence of vaccine serotypes 3 and 19A (poorly protected by PCV13) and highly virulent non-PCV13 serotypes due to replacement phenomena [10,11]. However, IPD rates fell considerably across the UK during the COVID-19 pandemic [12]. Conversely, we observed fewer *S. aureus* and Gram-negative infections compared with previous studies, reflecting national trends attributed to improved hospital infection control and stewardship measures [8,9, 13]. There was only one case of *Pseudomonas aeruginosa* empyema, which was hospital-acquired, which does not support empiric escalation from co-amoxiclav to piperacillin-tazobactam for community-acquired empyema.

Our findings reaffirm that microbial recovery through conventional culture methods of pleural tissue is poor (26.4% yield), due to both rapid sterilization of the pleural space by antibiotics and low sensitivity of culture for anaerobes. However, 16S rRNA PCR substantially improved diagnostic accuracy, particularly in culture-negative samples, detecting organisms in 68.2% of cases. Diagnostic yield was highest for *Streptococcus* species and anaerobes. 16S PCR was an effective antimicrobial stewardship tool, impacting prescribing in 12.5% of cases where it was used. However, turnaround times limited its potential impact as most results were unavailable until after hospital discharge. Positive 16S PCR results were available 10 days after surgery on average, which included time for transport of samples to the laboratory, days awaiting routine culture prior to 16S request, analytical and reporting time. Routine early use of 16S PCR (for example, for samples that are culture negative after 24 h) could maximize stewardship benefits. Whilst next-generation sequencing approaches (including Nanopore) have the potential to improve diagnostic sensitivity and better resolve polymicrobial infections compared with 16S sequencing, they are not a direct replacement. These methods require robust laboratory workflows, extensive validation and bioinformatics capacity and are not routinely available in most centres. In contrast, species-specific PCRs may offer greater sensitivity than broad-range 16S, but at the cost of breadth. The optimal molecular approach for pleural infection diagnostics, therefore, remains uncertain.

Rapid syndromic PCR-based panels have been proven to be effective stewardship tools in specific groups of patients with community and hospital-acquired pneumonia [14,15]. These platforms have demonstrated high sensitivity and specificity for key pathogens in respiratory samples, including *S. pneumoniae* [14,17]. An in-house syndromic PCR for community-acquired pleural infections was recently evaluated in a large comparative study. Its performance was evaluated against 16S. The syndromic PCR performed well and confirmed the diagnosis (i.e. detected at least one species) in 98.2% of cases and detected all present bacteria in 63.3%. Prospective evaluation of the syndromic PCR panel in the diagnostic routine showed that the sensitivity of the PCR for key empyema-causing pathogens greatly outperformed that of both culture and 16S [18]. Other studies in paediatric populations evaluated repurposing the rapid pneumococcal antigen test on pleural fluid and found a significant impact on antimicrobial decision-making [19]. [20], However, cross-reactivity with some oral streptococci, which have similar capsular polysaccharides, may potentially limit its utility in adults, given their high abundance in community-acquired pleural infection [5, 21]. Development of a syndromic PCR panel (either commercial or in-house) for pleural fluid or tissue would provide rapid diagnostic information and likely improve antibiotic use in empyema. Further prospective studies evaluating their utility in empyema are warranted. A drawback of molecular diagnostics is the lack of antimicrobial susceptibility data. In the UK, penicillin-resistant pneumococci are rare in the absence of recent international travel, supporting the safety of narrowing therapy based on molecular results. The oral pathobionts most frequently encountered also have very predictable susceptibility patterns, with the *S. anginosus* group normally susceptible to penicillin and the anaerobes susceptible to co-amoxiclav or metronidazole.

The optimal duration of antibiotics for empyema remains unclear, particularly in surgically managed cases. The British Thoracic Society recommends 2 to 6 weeks of therapy based on clinical response; however, there have been no randomized studies investigating optimal duration in surgical cohorts [4]. In our study, the median duration of antibiotics post-operatively was 14 days, shorter than durations reported in similar cohorts [13,22]. Microbiological diagnosis was associated with both longer antibiotic courses and longer hospital stays, consistent with previous studies and reflective of the severity of these cases [13]. Two small studies in medically managed pleural infection meeting pre-specified stability criteria both found that shorter courses of antibiotics (2–3 weeks vs. 4–6 weeks; 2 vs. 3 weeks) were not associated with treatment failure [23,24]. Prospective studies that look at shortened antibiotic courses for stable patients with thorough pleural cavity debridement would be valuable.

There are several limitations to this study. As a tertiary referral centre, most patients were transferred from other hospitals, limiting access to detailed data pertaining to comorbidity status, pre-transfer microbiological results and comprehensive follow-up data. Our study focused on surgically managed cases, introducing selection bias towards fitter patients. We did not collect detailed antimicrobial susceptibility data; however, Gram-negative organisms were uncommon. All *S. aureus* isolates were methicillin-sensitive, and all streptococci were sensitive to first-line agents.

This study provides insights into the epidemiology, microbial trends and clinical outcomes of surgically managed pleural empyema at a UK tertiary centre. Our findings highlight the importance of molecular diagnostics in improving pathogen detection and guiding antibiotic stewardship. Streamlining molecular diagnostics pathways or developing pleural-specific multiplex PCR panels could expedite bacterial identification and optimize antimicrobial therapy. Prospective studies are needed to evaluate shortened antibiotic regimens and to validate novel diagnostic platforms in pleural infection management.

## Supplementary appendices

Appendix S1. Study definition of broad and narrow spectrum antibiotic. For the purposes of this study, antibiotics considered to be broad-spectrum include penicillins containing β-lactamase inhibitors (e.g. amoxicillin/clavulanate), second through fifth generation cephalosporins, macrolides, quinolones, and lincomycin derivatives (e.g. clindamycin). Narrow-spectrum agents include penicillins (e.g. amoxicillin), first generation cephalosporins, glycopeptides, oxazolidinones (e.g. linezolid) and nitroimidazoles (e.g. metronidazole). Where combination therapy with two or more narrow-spectrum agents resulted in broad cover, this was classified as broad spectrum.

## Abbreviations

COVID-19: coronavirus disease 2019
IPD: invasive pneumococcal disease
SARS-CoV-2: severe acute respiratory syndrome coronavirus 2
VATS: video-assisted thoracoscopic surgery
